# Sirolimus-loaded CaP coating on Co-Cr alloy for drug-eluting stent

**DOI:** 10.1093/rb/rbw018

**Published:** 2016-04-27

**Authors:** Jingxin Yang, In-Seop Lee, Fuzhai Cui

**Affiliations:** ^1^Materials Science and Engineering, College of Mechanical and Electrical Engineering, Beijing Union University, Beijing 100020, China;; ^2^Beijing Engineering Research Center of Smart Mechanical Innovation Design Service, Beijing 100020, China;; ^3^Atomic-Scale Surface Science Research Center, Yonsei University, Seoul 120-749, Korea and; ^4^School of Materials Science and Engineering, Tsinghua University, Beijing 100084, China

**Keywords:** drug-eluting system, Co-Cr alloy, sirolimus

## Abstract

To achieve polymer-free and controllable drug-eluting system, there have been many efforts to modify the surface composition and topography of metal stent. Recently, calcium phosphate is commonly applied to metallic implants as a coating material for fast fixation and firm-implant bone attachment on the account of its demonstrated bioactive and osteoconductive properties. In the present study, the release of sirolimus could be controllable because of immobilization of sirolimus during the process of biomimetic CaP coating forming. A completely new concept is the drug carrier of biomimetic CaP coating with sirolimus for an absorbable drug eluting system, which in turn can serve as a drug reservoir. We here describe the characteristic, mechanisms and drug release in vitro of new drug-eluting system in comparison to conventional system equivalent. Nano-structured calcium phosphate (CaP) coating was formed on the cobalt–chromium (Co-Cr) alloy substrate. By immersing coated sample in solution with sirolimus (rapamycin), the sirolimus could be immobilized in the newly formed CaP layer. The morphology, composition and formation process of the coating were studied with scanning electron microscopy, energy dispersive spectrometer, X-ray diffraction and X-ray photoelectron spectroscopy. The results showed that a uniform CaP coating incorporated with sirolimus was observed on Co-Cr alloy.

## Introduction

Cobalt–chromium (Co-Cr) alloys occupy an important place as metallic biomaterials. Co-Cr alloys, according to ASTM standard, have been used for several decades [1] and recently have been used for making coronary stents. One of the important advantages for this material is to make ultra-thin struts with increased strength [2].

Currently, paclitaxel or sirolimus are used as the drug for available drug-eluting stents (DES). The earliest medical use for sirolimus is in 1975 as a macrolide antibiotic, which is also called rapamycin. Sirolimus has potent antifungal, immunosuppressant and antitumor properties [3, 4]. It is a potential therapeutic agent for the remedy of choroidal neovascularization and diabetic macular edema [5, 6]. One of the problems of metal surface for drug loading is that the surface is too smooth. However, porous surface provides the possibility of loading more drugs than the smooth metal surface because of the larger surface area and the stronger absorption ability. Drug delivery through biodegradation is the general method, and it has been extensively reviewed in the research for orthopedic [7], ocular [8] and cardiovascular applications [9]. Extensive research confirms a significantly reduction in the rate of restenosis compared to conventional bare metal stents [10]. At present, metal stent with polymer coatings is widely used. Nevertheless, it has been frequently reported that the traditional polymer coatings are not entirely inert, and hypersensitivity reactions against the polymer [11, 12]. Multiple data display long-term harmful effect, e.g. increased inflammation of the vascular wall, a thrombogenic response, and induced apoptosis of smooth muscle cells [13]. In order to reduce the inflammatory reaction and balance drug release, which is partially caused by the polymer, CaP coating is developed. It is good for extended release of sirolimus in DES. Biomimetic CaP coating is a proverbial and excellent bioceramic that is similar to biological apatite of bone [14, 15]. Otherwise, it is biocompatible, bioactive and bioresorbable [16]. The rate of degradation is controllable according to the morphology, structure and crystallinity [17, 18]. In addition, the porous structure of biomimetic CaP coating for the drug carrier is not dependent on the use of the polymer, which is named polymer-free drug-eluting system.

We here report on a novel drug-eluting system to control release of sirolimus. Kim *et al.* [19] reported Co-Cr alloys could not form a layer by means of NaOH treatment, and therefore did not form a CaP-coating layer in SBF. It is first time to form CaP coating on Co-Cr alloy by biomimetic method and immobilize sirolimus.

## Materials and methods

### Pre-treatment of Co-Cr substrate

The composition of Co-Cr alloy (Genoss Co, Korea) used in this study was ASTM 90, C 0.079, Si 0.18, *P* < 0.010, S <  0.002, Mn 1.20, Ni 10.57, Cr 20.52, Fe 0.69, Co BAL., W 14.80. Co-Cr alloy samples were chopped into disk-type samples with 2 mm of thickness and 10 mm of diameter. The surface of the samples was polished by 600# waterproof abrasive paper, and then was cleaned in acetone for 10 min, in alcohol for 10 min, in deionized water for 10 min by ultrasonic. And then all the discs were etched in mixed acid (HNO_3_:HF:H_2_O_2 _=_ _1:1:1) for 1 hour in ultrasonic bath. The cleaned Co-Cr alloy discs were batched for alkali treatment at a concentration of 1 M NaOH solution for 6 hours. The treatment temperature of 140°C was used in autoclave. Following this management, the samples were thoroughly washed with deionized water, and then were dried at 37°C in an electric oven.

### Preparation of the CaP coating

Biomimetic mineralization solution (BMS) was prepared by dissolving reagent grade CaCl_2_ (100 mg/L) in Dulbecco’s phosphate buffered saline (DPBS, Calcium/Magnesium free; Gibco-brl Life Technologies). Each sample was fully immersed into BMS solution at 37°C for 12 hours to form CaP coating.

For biodegradation test of CaP coating, the coated samples were immersed in a 10-ml normal saline solution at 37°C for up to 20 days in a water bath. Reagent grade NaCl (142 mM) was dissolved in ultra-pure water to prepare the normal saline, which is buffered to PH 7.4 at 37°C using TRIS (50 mM) and 1 M HCl.

### Immobilization of sirolimus

Sirolimus (rapamycin) was obtained from Genoss Co. For immobilization of sirolimus in the coating, each sample was incubated in solution at 37°C similar to the temperature of body. The samples were first immersed in BMS for 12 hours, and then incubated in a 5.0-ml solution with sirolimus concentration of 0.1 mg/ml for 12 hours. This solution containing siralimus was prepared by dissolving sirolimus into dimethyl sulphoxide, and then was diluted with 5-ml BMS. So SD is short for this solution containing siralimus. After incubation, all the samples were washed with deionized water, and then were dried at 37°C in an electric oven.

### Analysis of samples and solution

The sample surfaces were observed by scanning electron micros-copy (SEM, S-4200; Hitachi, Japan). At the same time, the Ca/P ratio of the CaP coating was investigated by energy dispersive spectrometer (EDS) equipment. X-ray diffraction (XRD; Rigaku, Tokyo) were used to evaluate the structure of the coating. In addition, X-ray photoelectron spectroscopy (XPS; PHI 5700, Netherlands) with Al Ka X-rays were used to analyze the compositional of samples before and after immersion into SD solution. The photoelectron take-off angle was set at 45°.

The concentration of sirolimus after soaking with samples was measured by high-performance liquid chromatography. The number of sirolimus was calculated on the basis of the reduced sirolimus concentration of the solution incubation with coatings. The changes of calcium ion (Ca^2+^) concentration at different time intervals were measured by a QuantiChrom™ calcium assay kit (DICA-500; BioAs-say Systems). In order to study, 200 µl of work reagent was added to aliquots of 5 µl of sample on 96-well plate. The plate was read at 595 nm using a micro-plate reader (Tecan Sunrise, Switzerland) at room temperature. A standard curve was set up by serial dilutions of CaCl_2_ (0–200 µg/ml). Depending on the concentration of Ca^2+ ^ in the solution, the reducing quantity of calcium could be determined. Certainly, it was assumed that the reduction of calcium in the solution was not deposited in any place except for the substrate.

## Result

### Morphology of coating

**Figure 1.**
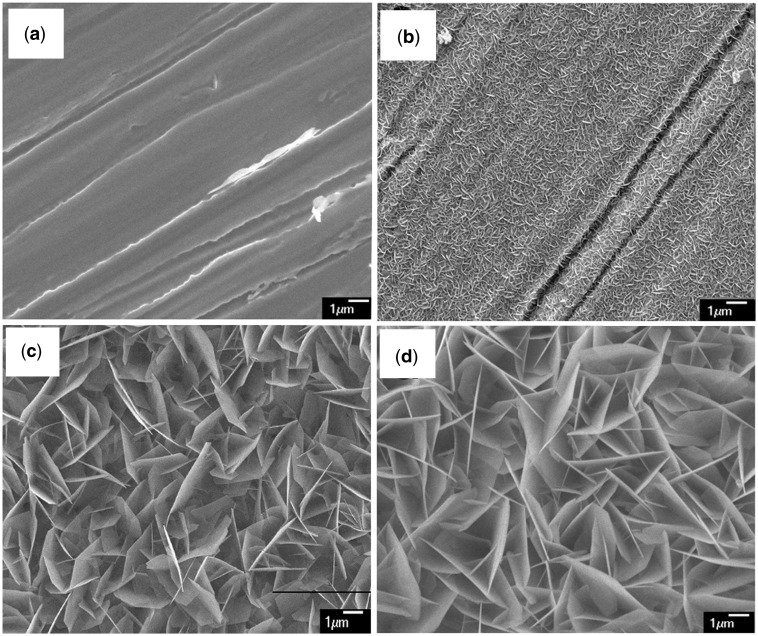
SEM morphologies of the surface of Co-Cr alloy (a) control substrate after etching by mixed acid, (b) after alkali-treatment, (c) immersion in BMS for 12 h (d) then incubation in SD solution for 12 hours.

Figure 1 shows the morphologies and the homological EDS analysis of the surface of Co-Cr alloy. The polished and etched surface by mixed acid is shown in Fig. 1a. As shown in Fig. 1b, a highly rough topography was obtained after alkali treatment, which was good for nucleation and the following growth of coating. Lamellate-like crystal morphologies shaped CaP coating was formed on the alloy after immersion in BMS for 12 hours (Fig. 1c). The crystals had <100 nm of thickness, ∼400 nm to 2 µm of width and around several micrometers of length. The composition of the coating was analyzed by EDS. Ca peak, P peak and O peak could be observed clearly. Moreover, the EDS results also showed the Ca/P ratios was ∼1.99 after 12-hour immersion, which was higher than that of HA (1.66) increasingly after prolonged immersion (Table 1). Typical morphology of biomimetic CaP coating retained after incubation in SD solution for 12 hours at a temperature of 37°C as shown in Fig. 1d.

**Table 1.** EDS results of the surface of Co-Cr alloy after immersion in BMS for 12 hours

Element	Mass%	Atom%
O	4.56	13.82
P	7.38	11.55
Ca	19.09	23.09
Cr	17.93	16.71
Co	38.24	31.45
M	12.80	3.37

### Composition and structure of the coatings

**Figure 2.**
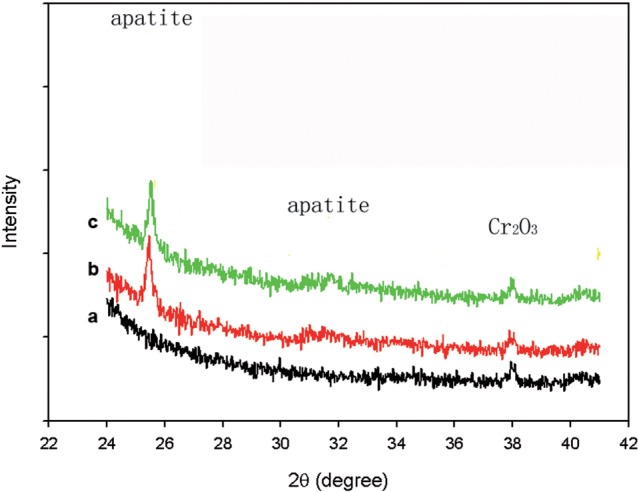
XRD patterns of the apatite coating formed on Co-Cr alloy (a) substrate control sample after alkali treatment (b), after immersion in BMS solution at 37 °C for 12-hour (c) incubation in SD solution for 12 hours.

The crystal structures of the coating were displayed by XRD patterns (Fig. 2). All the XRD patterns show peaks of Co-Cr alloy substrate. The CaP-coating crystal structure was showed on the other samples except the substrate control. Strong peaks near 25.3° and weaker ones near 32° were showed on both coated sample and sirolimus-incorporated sample corresponding to the reflection for CaP coating. Adsorption of sirolimus in SD solution for 12 hours did not affect crystal structure of CaP coating.

Based on the wide span of XPS spectra, the elemental compositions of surfaces before and after incubation in SD solution are shown in Table 2. Carbon is usually derived from unavoidable hydrocarbon contamination. The presentation of N contents reflected successful loading of sirolimus in the CaP coating as nitrogen exited only in sirolimus among the reagents used in this study. Nitrogen-to-carbon (N/C) ratio on immobilization in SD is 0.021, which is close to the theoretical N/C of 0.022 for sirolimus.

**Table 2.** Elemental compositional at the surface of coated samples before and after incubation in SD solution containing sirolimus determined by XPS

Surface	C%	N%	Ca%	P%
CaP coating	58	0	23.46	18.54
Immobilization in SD	76.66	1.58	11.93	9.83

Percentages computed based on the C, N, Ca and P contents only.

### Immobilization of sirolimus

#### Amount of sirolimus incorporated with samples

After incubation in SD solution for 12 hours, new formative well-distributed layer was noticed by SEM on the surface as shown in Fig. 1d. After the sample was incubated for 12 hours at 37°C, the concentrations of sirolimus in the solutions decreased obviously. Based on the above inference, the reduction of sirolimus in the solution was assumed to be completely incorporated with coatings. The amount of sirolimus incorporated with one sample in SD solution (total drug in solution is 500 μg) is 42.1 ± 7.4 μg. The ratio of successful drug loading reached >9.9%.

#### Changes of morphology after release

**Figure 3.**
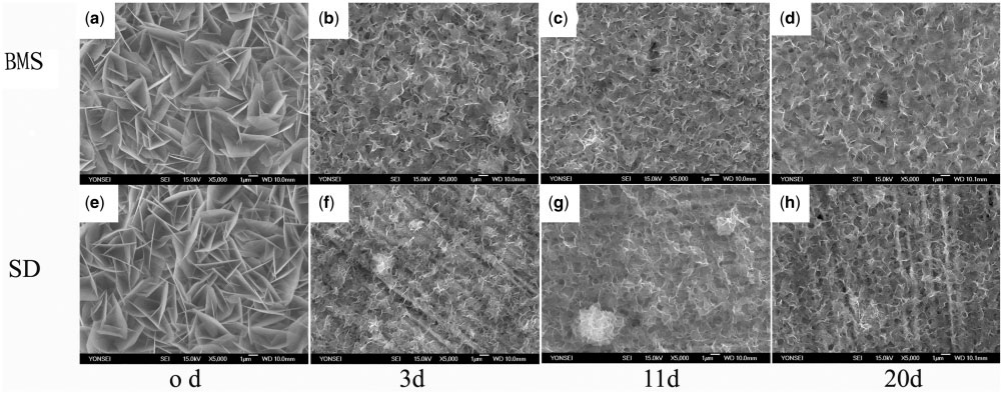
SEM micrographs of surfaces of samples after immersion in BMS for 3, 11 and 20 days at 37 °C. (a–d) Apatite coated samples, (e–h) incorporated with sirolimus after incubation in SD solution. All the scales are 1 μm in length.

Figure 3 shows the changes in morphology of the CaP coating and the sirolimus incorporated CaP coating after 3-, 11- and 20-day immersion in the normal saline solution. The changes show the biodegradation of the coating of the sample. The morphology of sirolimus-incorporated sample remained similar to that of only CaP-coating coated sample in physiological salt solution. Although the coating degraded, sirolimus was expected to release correspondingly.

## Discussion

### New drug-eluting system

It has been already proved that the formation of biomimetic coating on the surfaces of Si, Ti, Zr, Nb and Ta can be reproduced in supersaturated calcium phosphate solutions [20]. It is reported Co-Cr–Mo alloys could not form CaP coating in SBF. In this study, however, Co-Cr alloy was demonstrated to have CaP coating-forming abilities through alkali treatment and biomimetic process.

Due to the proven biocompatibility, bioactive and bioresorbable, combined with the ability to tune porosity, porous CaP coating now are explored for drug retention and elution. CaP coating is considered to be a valuable alternative to the polymer. The present XRD patterns show obvious coating structure. Ca/P ratios of EDS after 6 hours is higher than that of HA (1.66) and increase after prolonged immersion. The coating would be degraded in physiological fluids, and it was confirmed by the immersion test *in vitro* in the present study (Fig. 3). Furthermore, its microporous structure is beneficial for immobilization of sirolimus.

**Figure 4.**
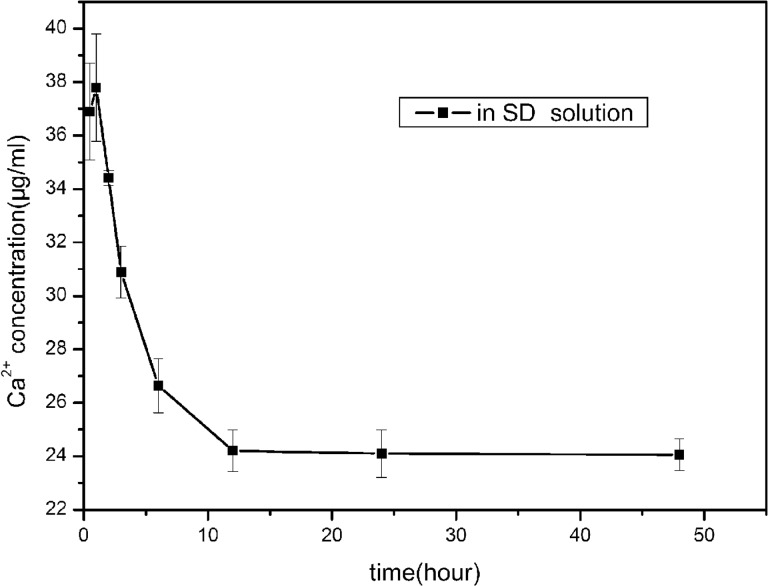
Ca^2+^ concentrations in SD solution for immersion after different time intervals. (0.5, 1, 3, 6, 12, 24, and 48 hours).

According to the result, the first 12 hours in BMS, it is the process for the growth of CaP coating on alloy. And the following 12 hours in SD solution, it is the process for the continuous growth of CaP coating with sirolimus encapsulation at the same time. Sirolimus was immobilized during the process of CaP coating forming. Sirolimus encapsulation was accompanied with CaP growth, not only through absorption, which is the outstanding of this new polymer-free drug-eluting system. As shown in Fig. 4, the sample incubated in the SD solution, Ca^2+ ^concentration initially increased to a maximum value 37.79 μg/ml after 1 hour, then began to decrease with incubation time, as calcium and phosphorus ions were deposited on the surfaces gradually as CaP coating. The maximum value of Ca^2+ ^concentration indicated the amount of Ca^2+ ^dissolved from the coating was larger than deposition onto the surface. Actually, Ca^2+ ^began to deposit meaningfully on the surface after the time corresponding to the highest point of the curve.

### Analysis of the coating

The crystals formed on the Co-Cr alloy were straight with sharp edges, altering from the morphology of nucleation to the morphology of growth after 12-hour immersion. The CaP-coating crystals formed with sirolimus on the coated surface were slightly curved and smaller. Furthermore, the morphology of nucleation did not change even after 12-hour incubation. Formation rate of CaP coating in BMS solution was faster than that in SD solution. The samples immersed in SD solution retained sharp edged lamellate-like crystal morphology and XRD patterns showed the CaP coating structure even incubation in SD solution. The sirolimus in the SD solution existed mainly as a free monomer or a complex with CaP coating. After a period of deposition, it is difficult to form new crystal in the original BMS solution. When the coated sample was incubated in SD solution, parts of the formed crystal served as apatite nuclei or precursors of apatite. Then new deposition, nucleation and spontaneous growth process would occur again. Because the SD solution is supersaturated relative to apatite and apatite has a lower solubility [21], the sirolimus is easily to be encapsulated and to form siolimus–apatite complexes. In the coprecipitation stage, sirolimus affected the growth and morphology of CaP coating. Therefore, the immobilization of sirolimus reduces the rate of CaP-coating formation, but may not inhibit CaP-coating formation.

The rate of biodegradation can be tuned by alkali treatment, and immersion time during the formation of CaP coating [17, 18]. In addition, the drug release profile can be controlled by adjusting the biological degradation of CaP coating. With this method, drug could release persistently and steadily.

CaP coating formed by the biomimetic technique serves as a delivery system to control the release of sirolimus on Co-Cr alloy. Therefore, when implants are used, sirolimus would be released and exert an influence as EDS.

## Conclusions

This study shows that (i) after immersion in BMS solution at 37°C, biomimetic CaP coating formed on the alkali-treated Co-Cr alloy substrate and (2) sirolimus was successfully immobilized on polymer-free and biomimetic CaP coating. The immobilization of sirolimus did not influence the morphology of the CaP-coating formation and biodegradation.

## References

[1] Brunski JB. Metals In: Ratner BD Hoffman AS Schoen FJ Lemons JE (ed). Biomaterials Science an Introduction to Materials in Medicine. 2nd edn San Diego: Elsevier Academic Press, 2004, 137–53

[2] Rittersma S Winter R Koch K Impact of strut thickness on late luminal loss after coronary artery stent placement. Am J Cardiol 2004;93:477–80. 14969629 10.1016/j.amjcard.2003.10.049

[3] Tanaka H Kuroda A Marusawa H Structure of FK-506 a novel immunosuppressant isolated from Streptomyces. J Am Chem Soc 1987;109:5031–3.

[4] Groth CG Bäckman L Morales JM Sirolimus (rapamycin)-based therapy in human renal transplantation: similar efficacy and different toxicity compared with cyclosporine. Transplantation 1999;67:1036–42. 10221490 10.1097/00007890-199904150-00017

[5] Paghdal KV Schwartz RA. Sirolimus (rapamycin): from the soil of Easter Island to a bright future. J Am Acad Dermatol 2007;57:1046–50. 17583372 10.1016/j.jaad.2007.05.021

[6] Jozwiak J Jozwiak S Oldak M. Molecular activity of sirolimus and its possible application in tuberous sclerosis treatment. Med Res Rev 2006;26:160–80. 16329102 10.1002/med.20049

[7] Saito N Murakami N Takahashi J Synthetic biodegradable polymers as drug delivery systems for bone morphogenetic proteins. Adv Drug Deliv Rev 2005;57:1037–48. 15876402 10.1016/j.addr.2004.12.016

[8] Yasukawa T Ogura Y Kimura H Drug delivery from ocular implants. Expert Opin Drug Deliv 2006;3:261–73. 16506952 10.1517/17425247.3.2.261

[9] Tanguay J Zidar J Phillips H Current status of biodegradable stents. Cardiol Clin 1994;12:699–713. 7850839

[10] Pasceri V Patti G Speciale G Meta-analysis of clinical trials on use of drug-eluting stents for treatment of acute myocardial infarction. Am Heart J 2007;153:749–54. 17452148 10.1016/j.ahj.2007.02.016

[11] Virmani R Guagliumi G Farb A Localized hypersensitivity and late coronary thrombosis secondary to a sirolimus-eluting stent: should we be cautious? Circulation 2004;109:701–5. 14744976 10.1161/01.CIR.0000116202.41966.D4

[12] Nebeker JR Virmani R Bennett CL Hypersensitivity cases associated with drug-eluting coronary stents: a review of available cases from the Research on Adverse Drug Events and Reports (RADAR) project. J Am Coll Cardiol 2006;47:175–81. 16386683 10.1016/j.jacc.2005.07.071

[13] Sousa JE Costa MA Farb A Images in cardiovascular medicine: vascular healing 4 years after the implantation of sirolimuseluting stent in humans: a histopathological examination. Circulation 2004;110:e5–6. 15238468 10.1161/01.CIR.0000134307.00204.B3

[14] Yang JX Cui FZ Yin QS Characterization and degradation study of calcium phosphate coating on magnesium alloy bone implant in vitro. IEEE Trans Plasma Sci 2009;37:1161–8. IF1.45

[15] Yang JX Cui FZ Lee I-S In vivo biocompatibility and degradation behavior of Mg alloy coated by calcium phosphate in a rabbit model. J Biomater Appl 2012;27:153–64. 21363872 10.1177/0885328211398161

[16] Joost D Patrick WS. Drug-eluting stent update 2007. Part I: a survey of current and future generation drug-eluting stents: meaningful advances or more of the same? Circulation 2007;116:316–28. 17638940 10.1161/CIRCULATIONAHA.106.621342

[17] Yang JX Cui FZ Jiao YP Calcium phosphate coating on magnesium alloy for modification of degradation behavior. Front Mater Sci 2008;2:143–8.

[18] Yang JX Jiao YP Yin QS Calcium phosphate coating on magnesium alloy by biomimetic method: investigation of morphology, composition and formation process. Front Mater Sci 2008;2:149–55.

[19] Kim HM Miyaji F Kokubo Preparation of bioactive Ti and its alloys via simple chemical surface treatment. J Biomed Mater Res 1996;32:409–17. 8897146 10.1002/(SICI)1097-4636(199611)32:3<409::AID-JBM14>3.0.CO;2-B

[20] Kokubo T Matsushita H Takadama T. Development of bioactive materials based on surface chemistry. J Eur Ceram Soc 2009;29:1267–74.

[21] Elliot JC. Structure and chemistry of the CaP coating and other calcium orthophosphates. Amsterdam: Elsevier Science BV; 1994, 1–61

